# Surgical treatment of hilar cholangiocarcinoma: retrospective analysis

**DOI:** 10.1093/bjsopen/zrad024

**Published:** 2023-05-17

**Authors:** Bin Li, Zhishuai Li, Zhiquan Qiu, Yingyi Qin, Qingxiang Gao, Jianyang Ao, Wencong Ma, Xiaoqing Jiang

**Affiliations:** Biliary Tract Surgery Department I, Eastern Hepatobiliary Surgery Hospital, Secondary Military Medical University, Shanghai, P. R. China; Biliary Tract Surgery Department I, Eastern Hepatobiliary Surgery Hospital, Secondary Military Medical University, Shanghai, P. R. China; Biliary Tract Surgery Department I, Eastern Hepatobiliary Surgery Hospital, Secondary Military Medical University, Shanghai, P. R. China; Department of Health Statistics, Second Military Medical University, Shanghai, P. R. China; Biliary Tract Surgery Department I, Eastern Hepatobiliary Surgery Hospital, Secondary Military Medical University, Shanghai, P. R. China; Biliary Tract Surgery Department I, Eastern Hepatobiliary Surgery Hospital, Secondary Military Medical University, Shanghai, P. R. China; Biliary Tract Surgery Department I, Eastern Hepatobiliary Surgery Hospital, Secondary Military Medical University, Shanghai, P. R. China; Biliary Tract Surgery Department I, Eastern Hepatobiliary Surgery Hospital, Secondary Military Medical University, Shanghai, P. R. China

## Abstract

**Background:**

Achieving a better prognosis for patients and reducing the risk of complications are primary considerations in surgical decisions for hilar cholangiocarcinoma.

**Methods:**

A retrospective analysis of the authors' clinical practice outcomes in the surgical management of patients with hilar cholangiocarcinoma following the planned-hepatectomy surgical treatment programme between 2009 and 2018.

**Results:**

Some 473 patients were included, of whom 127 (26.8 per cent) underwent bile duct tumour resection alone, 44 (9.3 per cent) underwent bile duct tumour resection combined with restrictive hepatectomy, and 302 (63.8 per cent) underwent bile duct tumour resection combined with extensive hepatectomy. R0 resection was achieved in 82.2 per cent and the postoperative complication rate was similar between the different operations. The 5-year survival rates after surgery were 37.0, 37.3, and 28.4 per cent in the bile duct tumour resection alone, restrictive hepatectomy, and extensive hepatectomy groups respectively, with no statistically significant differences. As TNM staging progressed, the 1–5-year cumulative survival rate for the patients in the three groups showed a significant downward trend.

**Conclusion:**

In the setting of a high-volume centre, a planned-hepatectomy surgical treatment programme helps to strike a better balance between achieving radical tumour resection for hilar cholangiocarcinoma and reasonable control of the extent of surgical damage.

## Teaser

Surgical safety and outcomes are the primary considerations in evaluating the rationale for surgical management of hilar cholangiocarcinoma. In order to effectively reduce the risk of surgery for hilar cholangiocarcinoma and increase the rate of radical tumour resection, we have developed a planned-hepatectomy surgical treatment programme that helps to strike a balance between achieving radical tumour resection and managing the extent of surgical damage, ensuring a better prognosis for patients and a lower risk of surgical complications.

## Introduction

Hilar cholangiocarcinoma (HCCA) is a small malignant tumour of the bile duct epithelium at the porta hepatis^1,2^ and is the most prevalent type of bile duct malignancy^3^. Because chemotherapy, targeted therapy, and immunotherapy have not made significant breakthroughs in the treatment of cholangiocarcinoma^3^, surgical treatment has been the most important clinical intervention for HCCA over the last 20 years^4–9^. Similarly, apart from surgery, there has been no major breakthrough in the value of chemotherapy or targeted agents for the adjuvant treatment of advanced or post-surgical HCCA. For cholangiocarcinoma that is not surgically resectable or has metastasized, chemotherapy regimens such as gemcitabine in combination with cisplatin, as well as gemcitabine in combination with S1, are the recommended chemotherapy regimens. However, the median survival for patients receiving these interventions is only about 12 months^10–12^. In the BILCAP study, which assessed the effectiveness and safety of adjuvant capecitabine for cholangiocarcinoma after surgery, patients in the HCCA group did not show a significant treatment effect^13^. Therefore, radical resection of the tumour through a standardized surgical programme remains a key factor in achieving optimal survival for HCCA patients today.

Surgical treatment of HCCA at the progressive stage often requires extensive hepatectomy (EH); however, if a patient undergoes surgery in a hyperbilirubinaemic state, the risk of postoperative complications, such as liver failure and haemorrhage, is high. To effectively screen high-risk patients for HCCA surgery, reduce the risk of such surgery, and increase the success rate of radical resection, the authors established a planned-hepatectomy surgical treatment programme for HCCA based on statistical analysis of treatment outcomes of their practice between 2000 and 2008. The clinical outcomes of 575 patients who were treated in accordance with this emergent planned-hepatectomy programme between 2009 and 2018 are summarized here.

## Methods

### Patients

**Fig. 1:**
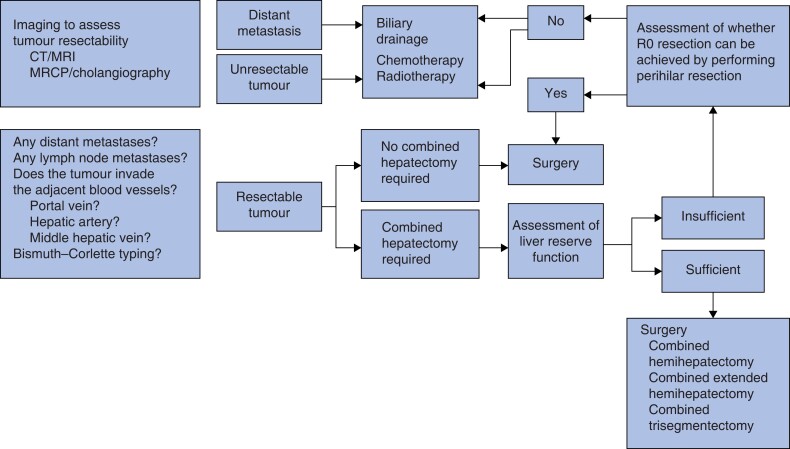
Imaging to assess tumour resectability CT, computed tomography; MRI, magnetic resonance imaging; MRCP, magnetic resonance cholangiopancreatography.

Data from 575 patients who underwent the surgical treatment between January 2009 and December 2018 were included in a prospective database. Patients were included for whom informed consent or a waiver of consent, along with follow-up information, were available. All patients who underwent surgery with the same surgery team in the Eastern Hepatobiliary Surgery Hospital were histologically diagnosed; however, patients presenting with intrahepatic cholangiocarcinoma or gallbladder cancer that had invaded the hilar bile duct were excluded from analysis. Additional exclusion criteria included: patients for whom informed consent was missing; and patients whose tumours were unable to be removed and who only underwent exploratory laparotomy or biliary drainage (*Fig. 1*).

None of the 575 patients received chemotherapy or radiotherapy before surgery. Adjuvant postoperative treatment was based on the following principles: for patients who had undergone R0 resection, S1 chemotherapy was routinely given for 3 courses; for patients who had undergone R1 resection, S1 chemotherapy in combination with platinum-based drugs was given for 6–12 courses, depending on liver function status and routine blood tests; and for patients who had undergone R2 resection or who had undergone dissection or biliary exploration, chemotherapy or radiotherapy was administered only in accordance with patient wishes and if permissible given liver function.

### Implementation scheme for the planned-hepatectomy surgical treatment programme

**Fig. 2:**
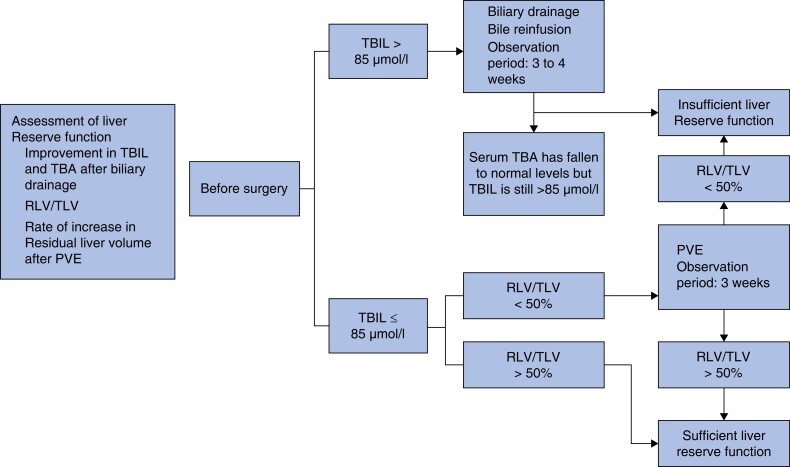
Assessment of liver reserve function TBIL, total serum bilirubin; TBA, total bile acids; RLV, residual liver volume; TLV, total liver volume; PVE, portal vein embolization.

**Fig. 3:**
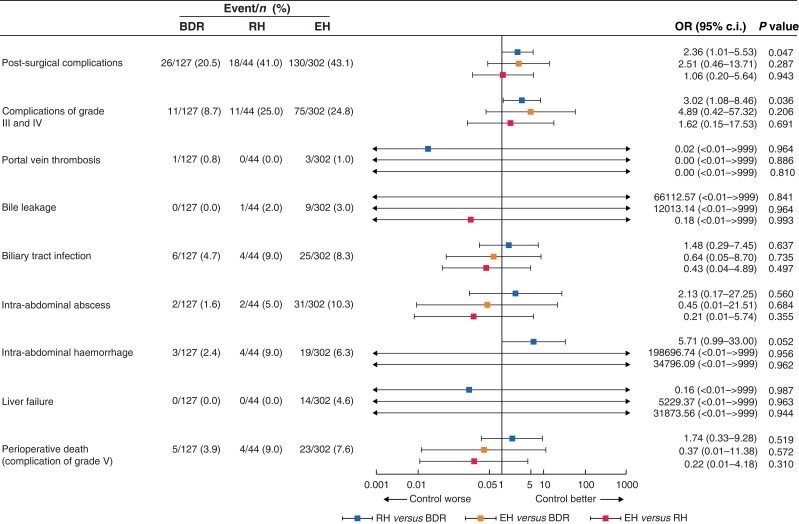
Analysisof major post-surgical complications and perioperative death in patients BDR, bile duct tumour resection alone; RH, restrictive hepatectomy; EH, extensive hepatectomy; OR, odds ratio.

In consideration of the known biological and clinical features of HCCA and considering the results of the authors' previous work, a planned-hepatectomy surgical treatment programme as outlined in *Fig. 2* and *Fig. 3* was implemented.

Based on the assessment of the imaging findings, patients who were surgical candidates were separated into three categories of surgical schemes: bile duct tumour resection alone (BDR), restrictive hepatectomy (RH), and EH. The RH group included patients receiving bile duct tumour resection combined with perihilar liver resection (liver resection of less than or equal to three segments^14^), whereas the EH group consisted of patients receiving bile duct tumour resection combined with hemihepatectomy and extended hemihepatectomy.

Accurate assessment of liver reserve function remains a clinical challenge, and there is no universally applicable protocol for assessing it across different liver disease states^15^. Although the indocyanine green clearance test is often used for this purpose, its accuracy can be severely disrupted by hyperbilirubinaemia^16,17^. Based on the results of our previous clinical practice and related studies^18^, the protocol for assessing the liver reserve function of patients in this study included: monitoring for a rapid decrease in total serum bilirubin (TBIL) after biliary drainage; and determining whether the resected liver volume (RLV) exceeded 50 per cent of the total liver volume (TLV) within 3 weeks of implementation of portal vein embolization (PVE). Due to the relatively slow progression of HCCA, prolonged biliary branch occlusion can trigger the pathophysiological phenomena of atrophy/proliferation syndromes in different liver segments^19^. The authors therefore used CT or MRI to accurately calculate the ratio of liver tissue volume in different regions to the TLV. During biliary drainage and PVE, a patient’s nutritional status was closely monitored, with nutritional support therapy provided as needed to maintain at least 75 per cent of the expected daily calorie and protein intake. For patients undergoing extracorporeal biliary drainage, bile reinfusion was performed to avoid the development of water and electrolyte imbalance. For patients with cholangitis due to pre-surgical biliary drainage, even if the patient’s bile bacterial culture was positive, the attending doctor approved the patient to proceed to the PVE or surgical treatment stage if the patient met the following criteria: normal blood leucocytes; negative blood bacterial culture results; and no febrile symptoms.

### Statistical analysis

Any complications occurring in patients receiving biliary drainage or PVE and surgical treatment, both during and for up to 90 days post-surgery, were recorded and rated for severity according to the Clavien–Dindo classification scheme^20^. Analysis focused on post-surgical liver failure, biliary leakage, intra-abdominal haemorrhage, intra-abdominal infection, and perioperative death, as these are the main adverse events that typically affect the perioperative clinical outcome of patients^21^.

Each patient was staged according to the staging criteria for HCCA of the UICC, TNM Classification of Malignant Tumours (8th edn)^22^. All enrolled patients were followed up regularly and their post-surgical survival status was recorded. The overall survival (OS) rates at 1, 3, and 5 years after surgery were calculated using the Kaplan–Meier method.

Summary statistics for patient demographic and clinical characteristics included calculated means and standard deviations (s.d.) for continuous variables, as well as frequencies and percentages for categorical variables. Comparisons among the BDR, RH, and EH groups were conducted using Student’s *t* tests, chi-squared tests, and Cochran–Mantel–Haenszel tests, as appropriate.

To compare OS among patients in the three groups, Kaplan–Meier curves were used to estimate survival functions, and hazard ratios (HRs) were calculated using both univariable and multivariable Cox proportional hazards models. Researchers also used the univariable and multivariable logistic regression models to compare rates of perioperative complications and perioperative deaths across the three groups.

Additionally, multivariable Cox or logistic regression models were used to evaluate rates of OS and post-surgical complications among patients in the BDR, RH, and EH groups who were further stratified according to relevant demographic and clinical characteristics. It is important to note that, for the TNM subgroup analysis, the following analytic approach was employed based on staging parameters and the number of actual patients available in each cohort. As both TNM stages I and II exclude the occurrence of tumour vascular invasion and lymph node metastasis, and as the number of stage I cases in this sample was small, stage I and II cases were combined and analysed together. Patients at TNM stage IIIa all experienced tumour vascular invasion, but no lymph node metastasis, and were, therefore, analysed independently. Furthermore, as the surgical protocols for TNM stages IIIb and IIIc are similar and given that most patients at both stages underwent R0 resection, these cases were combined and analysed together. Finally, as the number of cases at TNM stages IVa and IVb was small and given that most of these patients did not undergo R0 resection, they were combined to form an overall stage IV group.

Statistical analyses were performed using SAS software (version 9.4; SAS Institute Inc., Cary, NC, USA). All reported *P* values were derived from two-tailed tests, and a *P* value <0.050 was regarded as statistically significant. Figures were drawn using the R software package (version 3.5.2).

## Results

### Demographic and clinical characteristics and surgical treatment information for the full cohort

Of the 575 patients, 79 patients underwent only laparotomy or biliary drainage without tumour resection, and another 23 were lost to follow-up after surgery. The demographic and clinical characteristics of the final cohort of 473 patients are shown in *Table 1*. The median duration of the follow-up interval was 113 months.

**Table 1:** Demographic and clinical characteristics of patients undergoing surgical treatment

Characteristics	Total (*n* = 473)	Surgery scheme			
		BDR (*n* = 127)	RH (*n* = 44)	EH (*n* = 302)	*P*
**Sex**					0.172
Male	310 (65.5)	89 (70.1)	24 (54.6)	197 (65.2)	
Female	163 (34.5)	38 (29.9)	20 (45.5)	105 (34.8)	
**Age (years), mean(s.d.)**	59.63(9.5)	63.40(8.8)	62.02(8.5)	57.69(9.4)	<0.001
**TBIL before biliary drainage (μmol/l), mean(s.d.)**	171.99(136.6)	160.22(123.4)	211.34(129.9)	171.20(142.2)	0.100
**Pre-surgical biliary drainage**					0.005
No	163 (34.5)	60 (47.2)	9 (20.5)	94 (31.1)	
PTBD	269 (56.9)	55 (43.3)	32 (72.7)	182 (60.3)	
ERBD	28 (5.9)	9 (7.1)	0 (0.0)	19 (6.3)	
**Biliary T-tube drainage at another hospital**	4 (0.9)	1 (0.8)	1 (2.3)	2 (0.7)	
PTBD + ERBD	9 (1.9)	2 (1.6)	2 (4.6)	5 (1.7)	
**Pre-surgical TBIL value (μmol/l), mean(s.d.)**	87.97(81.5)	104.27(87.2)	125.07(121.6)	75.71(68.2)	<0.001
**Pre-surgical serum CA19-9 value (U/ml), mean(s.d.)**	346.52(363.5)	275.65(324.3)	351.76(406.2)	375.56(369.6)	0.007
<143.2	212 (44.8)	70 (55.1)	23 (52.3)	119 (39.4)	
≥143.2	261 (55.2)	57 (44.9)	21 (47.7)	183 (60.6)	
**Pre-surgical PVE**					<0.001
No	434 (91.8)	127 (100.0)	44 (100.0)	263 (87.1)	
Yes	39 (8.3)	0 (0.0)	0 (0.0)	39 (12.9)	
**Tumour type according to Bismuth–Corlette classification**					<0.001
I	105 (22.2)	83 (65.4)	22 (50.0)	0 (0.0)	
II	52 (11.0)	33 (26.0)	19 (43.2)	0 (0.0)	
III	95 (20.1)	10 (7.9)	1 (2.3)	84 (27.8)	
IV	221 (46.7)	1 (0.8)	2 (4.6)	218 (72.2)	
**Cut margin**					<0.001
R0	389 (82.2)	83 (65.4)	28 (63.6)	278 (92.1)	
R1	52 (11.0)	18 (14.2)	12 (27.3)	22 (7.3)	
R2	32 (6.8)	26 (20.5)	4 (9.1)	2 (0.7)	
Exploratory laparotomy	0 (0.0)	0 (0.0)	0 (0.0)	0 (0.0)	
**Combined caudate lobectomy**					<0.001
No	164 (34.7)	125 (98.4)	36 (81.8)	3 (1.0)	
Yes	309 (65.3)	2 (1.6)	8 (18.2)	299 (99.0)	
**Tumour vascular invasion**					0.468
No	324 (68.5)	94 (74.0)	34 (77.3)	196 (64.9)	
Portal vein	58 (12.3)	8 (6.3)	1 (2.3)	49 (16.2)	
Hepatic artery	44 (9.3)	14 (11.0)	6 (13.6)	24 (8.0)	
Portal vein and hepatic artery	47 (9.9)	11 (8.7)	3 (6.8)	33 (10.9)	
**UICC TNM stage**					<0.001
I and II	156 (33.0)	80 (63.0)	28 (63.6)	48 (15.9)	
IIIa	16 (3.4)	7 (5.5)	5 (11.4)	4 (1.3)	
IIIb and IIIc	283 (59.8)	33 (26.0)	10 (22.7)	240 (79.5)	
IV	18 (3.8)	7 (5.5)	1 (2.3)	10 (3.3)	
**Pathological tumour type**					0.912
Adenocarcinoma	458 (96.8)	123 (96.9)	42 (95.5)	293 (97.0)	
Squamous carcinoma	1 (0.2)	1 (0.8)	0 (0.0)	0 (0.0)	
Adenosquamous carcinoma	3 (0.6)	1 (0.8)	0 (0.0)	2 (0.7)	
Other types	11 (2.3)	2 (1.6)	2 (4.6)	7 (2.3)	
**Tumour cell differentiation grade**					0.083
High	11 (2.3)	4 (3.2)	2 (4.6)	5 (1.7)	
Moderate	439 (92.8)	113 (89.0)	36 (81.8)	290 (96.0)	
Low	23 (4.9)	10 (7.9)	6 (13.6)	7 (2.3)	
**Tumour peripheral nerve invasion**					0.024
No	127 (26.9)	39 (30.7)	18 (40.9)	70 (23.2)	
Yes	346 (73.2)	88 (69.3)	26 (59.1)	232 (76.8)	

Values are *n* (%) unless otherwise indicated. BDR, bile duct resection; RH, restrictive hepatectomy; EH, extensive hepatectomy; TBIL, total serum bilirubin; PTBD, percutaneous transhepatic biliary drainage; ERBD, endoscopic retrograde biliary drainage; CA19-9, carbohydrate antigen 19-9; PVE, portal vein embolization.

This cohort consisted of 310 (65.5 per cent) men and 163 (34.5 per cent) women, with a mean(s.d) age of 59.63(9.50) years for men and women combined. A total of 389 (82.2 per cent) patients underwent R0 resection, 52 (11.0 per cent) patients underwent R1 resection, and 32 (6.8 per cent) patients underwent R2 resection (*Table 1*).

Of the 473 patients, 127 (26.8 per cent) underwent BDR, 44 (9.3 per cent) underwent RH, and 302 (63.8 per cent) underwent EH. In the EH group, 46 patients received left hemihepatectomy, 44 received right hemihepatectomy, 144 received extended left hemihepatectomy, 66 received extended right hemihepatectomy, and 2 received right trisegmentectomy. Tumour vascular invasion occurred in 149 (31.5 per cent) of 473 patients, including 58 patients with invasion of the portal vein, 44 with invasion of the hepatic artery, and 47 with invasion of both. Reconstruction of the portal vein was performed in 54 patients.

Of the 50 patients who underwent combined resection of the residual hepatic lobar artery, the tumour was found to have invaded the right, left, or proper hepatic artery in 26, four, and nine patients respectively. In 11 patients, tumour invasion of both the lobar artery and the proper hepatic artery had occurred. Of these 50 patients, hepatic artery reconstruction of the residual liver lobe was performed in 14 patients.

The sex distribution was similar across the BDR, RH, and ER groups (men 70.1 *versus* 54.6 *versus* 65.2 per cent respectively, *P* = 0.172). The median age of patients in the BDR group was 63 (range 55–72) years, in the RH group was 62 (range 54–70) years, and in the EH group was 58 (range 48–67) years, with significant differences among the three groups (*P* < 0.001). There was no significant difference in TBIL among the three groups before biliary drainage or the number of patients undergoing surgery directly without biliary drainage (mean(s.d.) 160.22(123.37) *versus* 211.34(129.86) *versus* 171.20(142.16) µmol/l respectively, *P* = 0.100). However, there was a significant difference in carbohydrate antigen 19-9 (CA19-9) levels between the three groups, with patients in the EH group having higher levels than patients in either the BDR group or the RH group (mean(s.d.) 375.56(369.58) *versus* 275.65(324.33) *versus* 351.76(406.17) U/ml respectively, *P* = 0.034) (*Table 1*).

An R0 resection was achieved in 389 (82.2 per cent) patients. As shown in *Table 1*, the R0 resection rate was significantly higher in the EH group (92.1 per cent) than it was in the BDR group (65.4 per cent) and the RH group (63.6 per cent) (*P* < 0.001).

### Complications associated with pre-surgical biliary drainage and portal vein embolization

Of the 310 patients who received pre-surgical biliary drainage, four patients received surgical laparotomy and biliary drainage in other hospitals. Across the group, 219 patients received percutaneous transhepatic biliary drainage (PTBD) without liver failure or death, but two cases of bile leakage, one case of liver haemorrhage, and three cases of cholangitis occurred. A total of 37 patients underwent diagnostic endoscopy and biliary drainage at other hospitals or at the Gastrointestinal Disease Centre of the Eastern Hepatobiliary Surgery Hospital and, of these, five patients presented with significant cholangitis. An additional nine patients had an unsatisfactory reduction in TBIL after endoscopic retrograde biliary drainage (ERBD) such that remedial PTBD was required. For patients with combined cholangitis and fever, the pre-surgery treatment programme was to treat with targeted antibiotics with or without concomitant PTBD depending on the laboratory results (for example blood leucocytes, blood bacterial culture, and bile bacterial culture, etc.). When the patient’s febrile symptoms improved and the blood leucocyte and blood bacterial culture results returned to normal, antibiotic treatment was discontinued, and the surgical treatment was subsequently performed. All of these complications were classified as Clavien–Dindo grade I–II and did not result in a significant change in the clinical treatment programme. A total of 41 patients underwent pre-surgical PVE without any complications, such as bleeding, liver failure, or death. Two patients with PVE were found to have intra-abdominal metastasis of the tumour during operation, in which case the planned surgical scheme was abandoned and only bile duct drainage was performed.

### Post-surgical complications

The overall incidence of complications in the full cohort was 36.8 per cent (174/473), with an incidence of 20.5 per cent (97/473) of grade III or higher complications. The overall mortality rate within 90 days post-surgery was 6.8 per cent, with values of 3.9, 9.1, and 7.6 per cent in the BDR, RH, and EH groups respectively. There was no statistical difference in mortality rates across these three groups.

Of the 32 patients who died within 90 days of surgery, 14 had developed liver failure, including one with renal failure, one with respiratory and cardiac failure, and one with sepsis. Five deaths were due to intra-abdominal haemorrhage. One patient died because of gastrointestinal stress ulcer bleeding. One death was due to pulmonary infection combined with renal failure. Biliary leakage with intra-abdominal infection resulted in two deaths. Biliary tract infection complicated by sepsis resulted in four deaths. Acute myocardial infarction resulted in two deaths. Cardiac arrest resulted in two deaths. Finally, respiratory failure with heart failure resulted in one death.

Of the 36 patients in which arterial reconstruction was not performed, 14 (38.9 per cent) experienced no postoperative complications. However, complications occurred in 22 patients (61.1 per cent) post-surgery, including liver failure in five patients and pseudoaneurysm-related bleeding of the gastroduodenal artery in two patients. Six patients in this group died, including four due to liver failure, one due to ruptured haemorrhage from a pseudoaneurysm of the gastroduodenal artery, and one due to acute myocardial infarction. Of the 14 patients for whom arterial reconstruction was performed, seven had no postoperative complications (50 per cent), one died from hepatic haemorrhage, one died from severe abdominal infection, and the other five had Clavien–Dindo grade II complications. None of the patients who underwent hepatic artery reconstruction experienced postoperative liver failure.

The incidence of major post-surgery complications in the three groups is shown in *Fig. 3*. The only between-group differences occurred for patients with grade III complications, with those in the RH group (25.0 per cent) experiencing more complications relative to those in the BDR group (8.7 per cent, *P* = 0.036). Post-surgery mortality rate was higher for patients in the RH group (9.1 per cent) compared with those in the EH group (7.6 per cent) and in the BDR group (3.9 per cent). However, multifactorial analysis showed that these differences were not statistically significant (*Fig. 3*).

### Overall survival

**Fig. 4:**
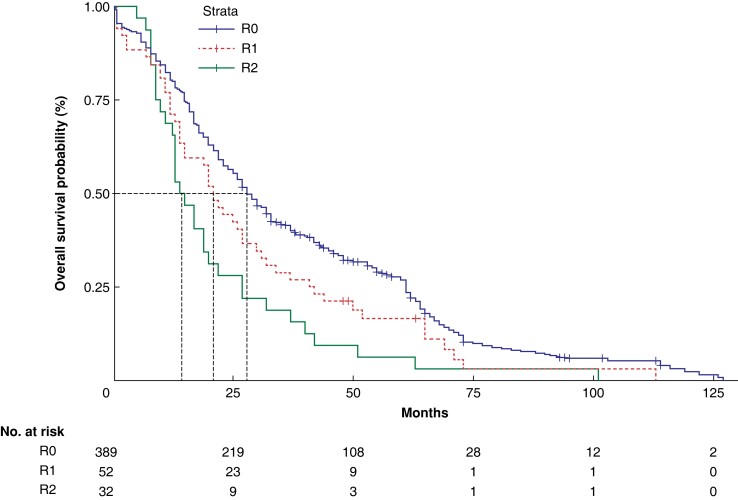
Overall survival analysis of patients with R0, R1, and R2 resection

The 1-, 3-, and 5-year post-surgical OS rates for the 473 patients in the full cohort were 78.4, 38.6, and 24.2 per cent respectively. The median OS duration for patients with R0, R1, and R2 resection was 28, 21, and 14.5 months respectively, with 5-year post-surgical OS rates of 26.8, 16.5, and 6.3 per cent respectively (*Fig. 4*).

The median survival duration after surgery was 26, 29, and 27 months for patients in the BDR, RH, and EH groups respectively. The 5-year post-surgery OS rates were 25.0, 28.2, and 23.4 per cent respectively (*Table S1* and *Fig. S1*). Univariable and multifactorial analyses of OS showed no statistically significant differences among the three groups (*Table S2*).

### Subgroup analyses

Subgroup analyses of post-surgery complications and OS in the BDR, RH, and EH groups are displayed in *Table 2*.

**Table 2:** Subgroup analysis of post-surgical complications and perioperative mortality rate

Subgroup	BDR	RH	EH	RH *versus* BDR		EH *versus* BDR		EH *versus* RH	
				OR (95% c.i.)	*P*	OR (95% c.i.)	*P*	OR (95% c.i.)	*P*
**Age (years)**									
<60	3/39 (7.7)	2/8 (25.0)	38/157 (24.2)	12.06 (0.96,151.17)	0.054	55 948.50 (0.00,3.74E153)	0.950	4640.40 (0.00,3.12E152)	0.962
≥60	8/88 (9.1)	9/36 (25.0)	37/145 (25.5)	4.02 (1.15,14.09)	0.030	2.69 (0.16,44.49)	0.489	0.67 (0.05,9.39)	0.766
**Pre-surgical biliary drainage**									
No	6/60 (10.0)	2/9 (22.2)	19/94 (20.2)	4.60 (0.50,42.26)	0.178	595 056.39 (0.00,2.51E136)	0.931	129 414.86 (0.00,5.45E135)	0.939
Yes	5/67 (7.5)	9/35 (25.7)	56/208 (26.9)	2.87 (0.71,11.64)	0.140	3.19 (0.21,47.82)	0.402	1.11 (0.09,13.83)	0.935
**Pre-surgical serum CA19-9 (U/l)**									
<143.2	7/70 (10.0)	6/23 (26.1)	22/119 (18.5)	4.79 (0.91,25.09)	0.064	0.50 (0.01,51.37)	0.769	0.10 (0.00,8.95)	0.320
≥143.2	4/57 (7.0)	5/21 (23.8)	53/183 (29.0)	8.97 (1.53,52.41)	0.015	160 184.25 (0.00,3.24E126)	0.933	17 862.73 (0.00,3.60E125)	0.945
**PVE**									
No	11/127 (8.7)	11/44 (25.0)	62/263 (23.6)	3.25 (1.16,9.12)	0.026	5.23 (0.43,63.05)	0.193	1.61 (0.15,17.92)	0.698
**Bismuth-Corlette type**									
III and IV	0/11 (0.0)	1/3 (33.3)	75/302 (24.8)	1.50 (<0.01,>999.99)	0.999	1.00 (<0.01,>999.99)	1.000	0.67 (0.05,9.71)	0.767
**Positive resection margin**									
R0	6/83 (7.2)	5/28 (17.9)	70/278 (25.2)	3.25 (0.80,13.22)	0.010	3.27 (0.95,11.21)	0.060	1.01 (0.27,3.73)	0.994
R1	4/18 (22.22)	3/12 (25.0)	5/22 (22.7)	0.00 (0.00,9.48E21)	0.670	0.00 (0.00,1.17E80)	0.700	0.00 (0.00,3.20E81)	0.781
**Tumour vascular invasion**									
No	7/94 (7.5)	5/34 (14.7)	48/196 (24.5)	1.98 (0.47,8.31)	0.351	0.00 (0.00,2.40E128)	0.950	0.00 (0.00,1.21E128)	0.947
Yes	4/33 (12.12)	6/10 (60.0)	27/106 (25.5)	9.73 (0.99,95.66)	0.051	15 209.48 (0.00,8.52E80)	0.915	1562.77 (0.00,8.65E79)	0.935
**Combined caudate lobectomy**									
Yes	0/2 (0.0)	0/8 (0.0)	74/299 (24.8)	0.99 (0.00,1.01E134)	1.000	19 106.47 (0.00,3.04E184)	0.963	19 240.60 (0.00,5.77E124)	0.944
**UICC TNM stage**									
I and II	7/80 (8.8)	3/28 (10.7)	14/48 (29.2)	2.83 (0.54,14.80)	0.218	162 058 040.00 (0.00,4.09E180)	0.926	57 306 474.70 (0.00,1.44E180)	0.930
IIIa	1/7 (14.3)	4/5(80.0)	1/4 (25.0)	0.00 (<0.01,>999.99)	0.957	0.00 (<0.01,>999.99)	0.949	6.29 (0.00,2.28E295)	0.996
IIIb and IIIc	3/33 (9.1)	4/10 (40.0)	57/240 (23.8)	5.36 (0.36,79.65)	0.223	3.61 (0.10,137.80)	0.490	0.67 (0.05,10.01)	0.774
IV	0/7 (0.0)	0/1 (0.0)	3/10 (30.0)	0.51 (<0.01,>999.99)	0.999	1.53E13 (<0.01,>999.99)	0.980	2.999E13 (<0.01,>999.99)	0.971
**Tumour peripheral nerve invasion**									
No	2/39 (5.1)	4/18 (22.2)	19/70 (27.1)	16.95 (0.69,416.08)	0.083	79 799.75 (0.00,4.36E126)	0.937	4707.83 (0.00,2.53E125)	0.953
Yes	9/88 (10.2)	7/26 (26.9)	56/232 (24.1)	2.49 (0.71,8.75)	0.155	2.94 (0.21,40.41)	0.420	1.18 (0.09,15.26)	0.898

Values are event/*n* (%) unless otherwise indicated. RH, restrictive hepatectomy; BDR, bile duct resection; EH, extensive hepatectomy; OR, odds ratio; CA19-9, carbohydrate antigen 19-9; PVE, portal vein embolization; UICC, Union for International Cancer Control.

For patients greater than or equal to 60 years of age and for whom pre-surgical serum CA19-9 levels were greater than or equal to 143.2 U/l and no PVE was performed, post-surgery complications were higher in the RH group than in the BDR group (*P* < 0.050). As shown in *Table 2*, in the various demographic and clinical subgroups, there were no statistically significant differences in the incidence of postoperative complications or perioperative mortality rate among patients in the BDR, RH, and EH groups (*P* > 0.050).

**Fig. 5:**
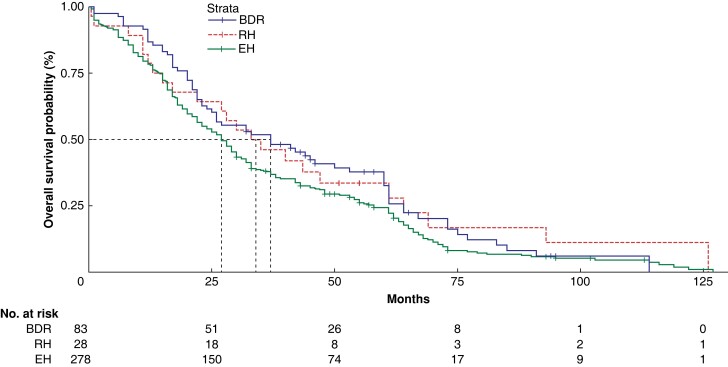
Overall survival analysis between the BDR, RH, and EH groups in patients with R0 resection BDR, bile duct tumour resection alone; RH, restrictive hepatectomy; EH, extensive hepatectomy.

In all the subgroups, there was no statistically significant difference in OS among patients who underwent BDR, RH, and EH (*P* > 0.050). There was also no statistically significant difference in survival among these groups for patients with R0 resection (*Fig. 5*). The median survival duration for patients in the three groups was 37, 34, and 27 months respectively, with 5-year post-surgery OS rates of 32.6, 33.6, and 24.4 per cent respectively.

**Fig. 6:**
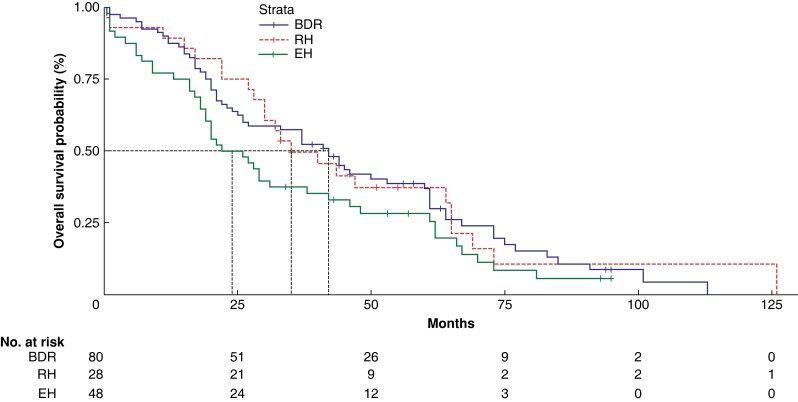
Overall survival analysis of patients at TNM stages I and II BDR, bile duct tumour resection alone; RH, restrictive hepatectomy; EH, extensive hepatectomy.

**Fig. 7:**
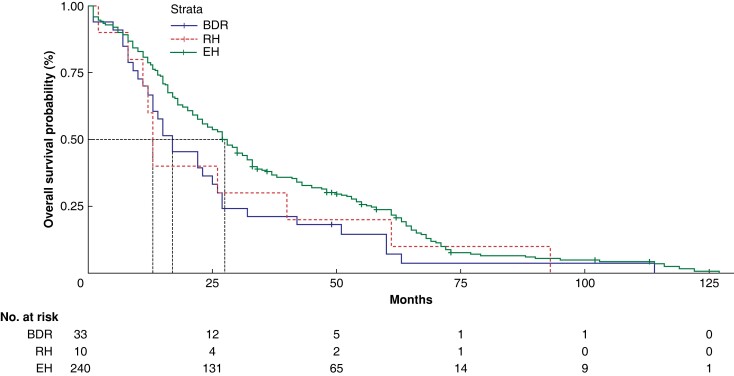
Overall survival analysis of patients at TNM stages IIIb and IIIc BDR, bile duct tumour resection alone; RH, restrictive hepatectomy; EH, extensive hepatectomy.

Finally, although the OS time after surgery in the full cohort decreased as the TNM stage increased, there was no statistically significant difference in survival across patients who underwent different surgical schemes within each specific TNM stage (*Fig. 6*, *Fig. 7*, *Table S3*, *Fig. S2*, and *Fig. S3*).

## Discussion

Surgical treatment is still the most effective treatment for HCCA^23^. Various staging systems for HCCA are currently used clinically as the main basis for decision-making in surgical planning^19,24,25^, with the Bismuth–Corlette classification being the most widely used surgical planning system for HCCA. The prevailing clinical opinion remains that BDR is sufficient for patients with Bismuth–Corlette type I and that it should be combined with caudal lobectomy for patients with Bismuth–Corlette type II. For Bismuth–Corlette type III and some type IV patients, combination with hemihepatectomy or extended hemihepatectomy is usually recommended, as a more thorough resection of the tumour can be obtained by removing more liver tissue.

It is generally accepted that the thoroughness of tumour resection is the most important factor affecting the prognosis of patients with HCCA, but there are differing views on the appropriate extent of surgery for patients with various states of disease progression. It has been suggested that EH should be performed in patients with Bismuth–Corlette types I and II^26–29^. However, an analysis of multicentre data from China and the USA showed that EH (left or right hemihepatectomy) was associated with a higher rate of serious postoperative complications compared with BDR. In well-selected patients with Bismuth–Corlette types I and II, the R0 resection rate, OS, and recurrence-free survival rate were comparable for patients receiving either BDR or EH^30^. As EH involves a higher risk of surgical trauma to the patient, several authors have advocated that RH should be performed instead for Bismuth–Corlette type III and IV patients^31–33^. As the ability to achieve a negative tumour cut margin is an important factor influencing surgical decisions, it is particularly important to clarify the criteria by which to define an R0 resection and its clinical significance. Greater clarity will help to resolve decision-making controversies and develop a rational surgical programme.

Concerning the diagnostic criteria for pR0/pR1 at the pathological bile duct cut margin for HCCA^34^, the International Collaboration for Cancer Reporting (ICCR) recommends that both radial and transverse pR0 of the bile duct margins should be greater than or equal to 1 mm^35^, but this definition draws only upon the results of studies of pancreatic cancer with similar pathological characteristics and is, therefore, not supported by extensive research evidence. More studies have used microscopic absence of tumour cells (greater than 0 mm) at the bile duct cut margin as a pR0 criterion^36^ and have confirmed that its prognosis remains significantly better than that of pR1^9,37^. Although there is a view that cholangiocarcinoma tends to grow in the submucosal and mucosal layers, it has been shown that bile duct narrow cut margins (less than or equal to 5 mm) and wide cut margins (greater than 5 mm) are not associated with survival differences in the patients with R0 resection^38^. In this study it was the microscopic absence of tumour cells (greater than 0 mm) at the bile duct cut margin that was used as a criterion for pR0 and as an important basis for decision-making on the extent of combined liver resection.

Reasons for the low rate of R0 resection in Bismuth–Corlette type II without tumour vascular invasion were related to the pre-surgery imaging diagnosis of Bismuth–Corlette type I and the implementation of BDR. This result suggests that it is difficult to differentiate between Bismuth–Corlette types I and II based on conventional imaging, especially when the bile duct branches of the caudate lobe are not significantly dilated. This may be since cholangiocarcinoma displays a subtle growth pattern along the mucosal layer of the duct wall and is difficult to detect effectively with conventional imaging techniques. Although routine intraoperative pathological examination of the bile duct cut margin can prevent misdiagnosis, the more complex anatomical features of the caudate lobe make it debatable whether such pathological examination should be routinely performed. This is because, even if the pathology of the bile duct cut margin of the caudate lobe is negative, the caudate lobe will still need to be removed in order to minimize risk of post-surgery biliary leakage, which would make the procedure significantly more difficult.

In this cohort, the overall mortality rate within 90 days of surgery was lower than that reported in the literature (1.4 to 18.0 per cent during hospitalization or 90 days after surgery)^39–48^. However, failure to reconstruct after hepatic artery resection significantly increased the risk of postoperative liver failure and death. HCCA is prone to tumour invasion of the hepatic artery, and the success of arterial reconstruction is limited both by the extent of tumour invasion and the difficulty of the operation. Furthermore, there is some debate about the need for revascularization^49–51^. The results of this study suggest that reconstruction of the hepatic artery in the remnant liver is important to reduce the risk of serious postoperative complications in HCCA.

In this study, OS time was significantly longer for patients in the R0 resection group than it was for those in the R1 and R2 resection groups. In the R0 resection group, there was no difference in OS rates at 1, 3, and 5 years post-surgery after BDR, RH, or EH. As the prognosis for patients with HCCA may be governed by population distribution, surgical treatment philosophy and strategy, and biological characteristics of the tumour, previous studies have tended to report mixed results. In 1997, Klempnauer *et al*.^52^ reported that patients undergoing BDR have a better prognosis than patients undergoing EH (31.8 *versus* 24.2 per cent respectively). The majority of studies with more than 150 cases report that the prognosis of patients who receive BDR is inferior to that of patients who receive EH. For example, the 5-year post-surgery survival rate after BDR is 0–18.0 per cent, whereas that of EH is 26.6–42.0 per cent^39,41,44–46,48^. In 2019, Tran *et al*.^5^ also reported higher 5-year survival rates after EH relative to BDR (8.8 *versus* 3.1 per cent respectively) for 194 patients with HCCA across 10 academic medical centres in the USA between 2000 and 2015.

Bismuth *et al*.^24^ concluded that negative bile duct cut margin is the most important factor affecting the prognosis of patients with HCCA and that, compared with other gastrointestinal cancers, the occurrence of lymph node metastasis in HCCA is not a critically adverse parameter affecting prognosis. The results of the Nagoya University study, however, suggest that lymph node metastasis is an important risk factor affecting the prognosis of patients with HCCA^47^. Similar to the results of a previous multicentre study published by the current authors^9^, this study reaffirms that biological factors, such as pre-surgical serum CA19-9 levels, tumour invasion of blood vessels, and lymph node metastasis, are all key risk factors affecting patient prognosis.

Despite its strengths, the study has some limitations to consider. First, this study is a single-centre, retrospective, observational study, and the clinical outcomes of the patients in the cohort were inevitably influenced by the treatment programmes that our team administered to patients. Considering this, the findings presented here need to be corroborated with those from other centres. Second, although the authors applied a multivariable regression model and propensity score methods to control for the effects of confounders, the results might have been influenced by unmeasured variables (for example post-surgery supportive treatment), thus introducing potential bias into the data.

Because the biology of cholangiocarcinoma and the symptoms of jaundice trigger complex pathophysiological changes in a patient’s physical status, surgical treatment decisions for HCCA need to strike a balance between achieving radical tumour resection on the one hand and reasonably controlling the degree of surgical damage on the other. Such a balance will help ensure that patients can achieve a better prognosis with a lower risk of surgical complications. The authors' established protocol and intervention pathway for planned hepatectomy in HCCA provide useful assistance to clinicians to achieve these important goals.

## Supplementary Material

zrad024_Supplementary_Data

## Data Availability

The authors declare that relevant data supporting the findings of this study are available within the paper and *Supplementary material*. Due to ethical and privacy concerns, they are unable to publish individual-level data in this study. Raw data on demographics, clinical characteristics, surgical treatment, and prognostic information for the overall cohort are available from the corresponding authors upon reasonable request.
