# Correction: Surgical treatment of hilar cholangiocarcinoma: retrospective analysis

**DOI:** 10.1093/bjsopen/zrad068

**Published:** 2023-06-23

This is a correction to: Bin Li and others, Surgical treatment of hilar cholangiocarcinoma: retrospective analysis. *BJS Open* 2023;**7**: https://doi.org/10.1093/bjsopen/zrad024

In the originally published version of this article, there were errors in the use of ‘>’ and ‘<’ symbols in the RLV/TLV presentation of the site area in *Fig. 2*. This is the correct version:

**Fig. 2:**
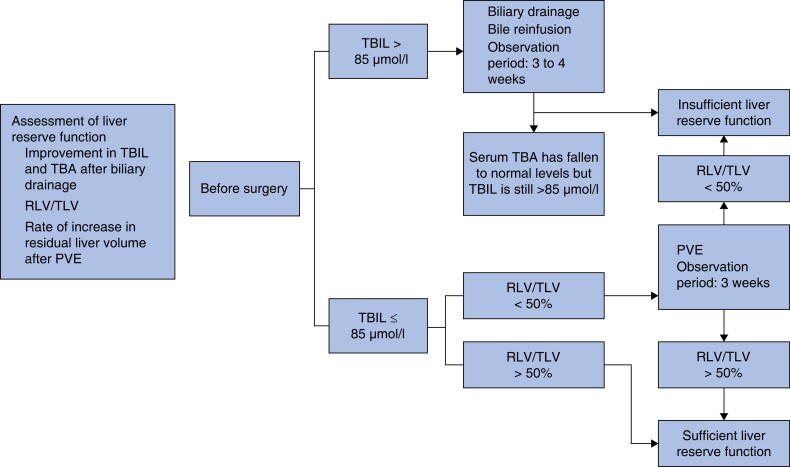
Assessment of liver reserve function

TBIL, total serum bilirubin; TBA, total bile acids; RLV, residual liver volume; TLV, total liver volume; PVE, portal vein embolization.

instead of:

**Fig. 2:**
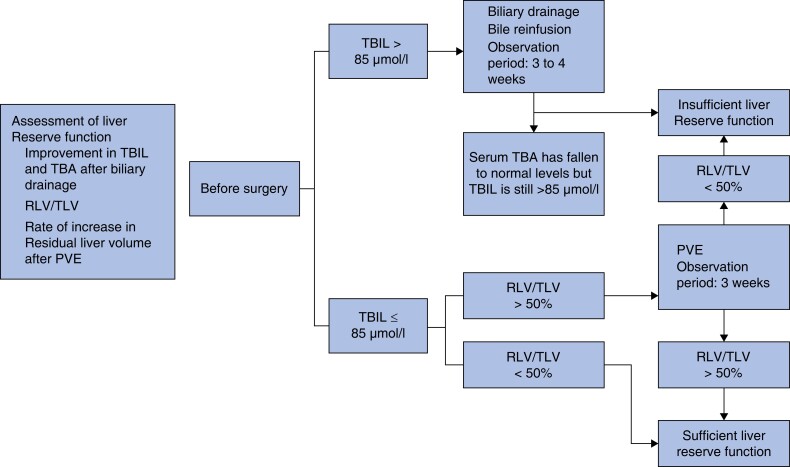
Assessment of liver reserve function

TBIL, total serum bilirubin; TBA, total bile acids; RLV, residual liver volume; TLV, total liver volume; PVE, portal vein embolization.

The figure has been emended in the article.

