# Supplementary material for: Surgical treatment of hilar cholangiocarcinoma: retrospective analysis
Source: BJS Open. 2023 May 17;7(3):zrad024. doi: 10.1093/bjsopen/zrad024 (PMC10189280; doi:10.1093/bjsopen/zrad024)
Supplement: zrad024_Supplementary_Data [file zrad024_supplementary_data.doc]

**TItle**

Surgical treatment of hilar cholangiocarcinoma: retrospective analysis of 473 resected patients

Author: Bin Li^1^*, Zhishuai Li^1^*, Zhiquan Qiu^1^*, Yingyi Qin^2^*, Qingxiang Gao^1^, Jianyang Ao^1^ ,Wencong Ma^1^, Xiaoqing Jiang^1^

1 Biliary Tract Surgery Department I, Eastern Hepatobiliary Surgery Hospital, Secondary Military Medical University

2 Department of Health Statistics, Second Military Medical University

**Corresponding author.** Xiaoqing Jiang,, No. 225 Changhai Road, Shanghai, 200438, P. R. China.

**Supplementary Materials - Index**

| **Supplementary Figures and Tables** |  |
| --- | --- |
| Table S1 | *pag. 2* |
| Table S2 | *pag. 3* |
| Table S3 | *pag. 4* |
| Figure S1 | *pag. 5* |
| Figure S2 | *pag. 6* |
| Figure S3 | *pag. 7* |

**Supplementary Figures and Tables**

**Table S1 Cumulative survival 1 to 5 years after surgery**

in the BDR, RH and EH groups

| Year | BDR | RH | EH |
| --- | --- | --- | --- |
| 1 | 80.3(73.7-87.5) | 77.3(65.8-90.7) | 77.8(73.3-82.6) |
| 2 | 52.0(44.0-61.4) | 56.8(43.9-73.5) | 53.0(47.6-58.9) |
| 3 | 42.5(34.7-52.0) | 38.5(26.5-56.0) | 36.9(31.9-42.8) |
| 4 | 31.2(24.0-40.7) | 28.2(17.4-45.8) | 28.8(24.1-34.5) |
| 5 | 24.9(18.1-34.4) | 28.2(17.4-45.8) | 23.4(18.9-28.8) |

Abbreviations: BDR, bile duct resection; RH, restrictive hepatectomy; EH, extensive hepatectomy.

**Table S2 Analysis of overall survival differences**

**between** BDR, RH and EH groups

| Statistical comparison | HR | 95% CI | *P* value |
| --- | --- | --- | --- |
| Univariate analysis |  |  |  |
| RH vs BDR | 0.959 | 0.664-1.384 | 0.8232 |
| EH vs BDR | 1.063 | 0.852-1.327 | 0.5870 |
| EH vs RH | 1.109 | 0.792-1.552 | 0.5476 |
| Multifactor analysis |  |  |  |
| RH vs BDR | 1.001 | 0.654-1.531 | 0.9973 |
| EH vs BDR | 1.316 | 0.605-2.864 | 0.4886 |
| EH vs RH | 1.315 | 0.592-2.924 | 0.5015 |

Abbreviations: BDR, bile duct resection; RH, restrictive hepatectomy; EH, extensive hepatectomy; HR, hazard ratio; CI, confidence interval.

**Table S3 Overall survival rate of patients with BDR, RH and EH in subgroup of TNM stage**

| Surgery scheme | TNM Stage | | | |
| --- | --- | --- | --- | --- |
|  | I and Ⅱ | Ⅲa | Ⅲb and Ⅲc | IV |
| BDR(%) |  |  |  |  |
| 1 year | 87.5(80.5-95.1) | 71.4(44.7-100.0) | 66.7(52.4-84.9) | 71.4(44.7-100.0) |
| 3 year | 57.5(47.6-69.4) | 14.3(2.3-87.7) | 21.2(11.0-40.9) | 0.0 |
| 5 year | 37.0(27.3-50.1) | 0.0 | 7.3(2.0-26.5) | 0.0 |
| RH(%) |  |  |  |  |
| 1 year | 89.3(78.5-100.0) | 60.0(29.33-100.0) | 60.0(36.2-99.5) | 0.0 |
| 3 year | 49.7(34.2-72.3) | 0.0 | 30.0(11.6-77.3) | 0.0 |
| 5 year | 37.3(22.7-61.3) | 0.0 | 20.0(5.79-69.08) | 0.0 |
| EH(%) |  |  |  |  |
| 1 year | 77.1(66.1-89.9) | 50.0(18.8-100.0) | 78.8(73.7-84.1) | 70.0(46.7-100.0) |
| 3 year | 37.5(26.0-54.0) | 25.0(4.6-100.0) | 38.1(32.4-44.8) | 10.0(1.6-64.2) |
| 5 year | 28.4(18.0-44.8) | 0.0 | 23.8(18.9-30.1) | 0.0 |

Abbreviations: BDR, bile duct resection; RH, restrictive hepatectomy; EH, extensive hepatectomy.


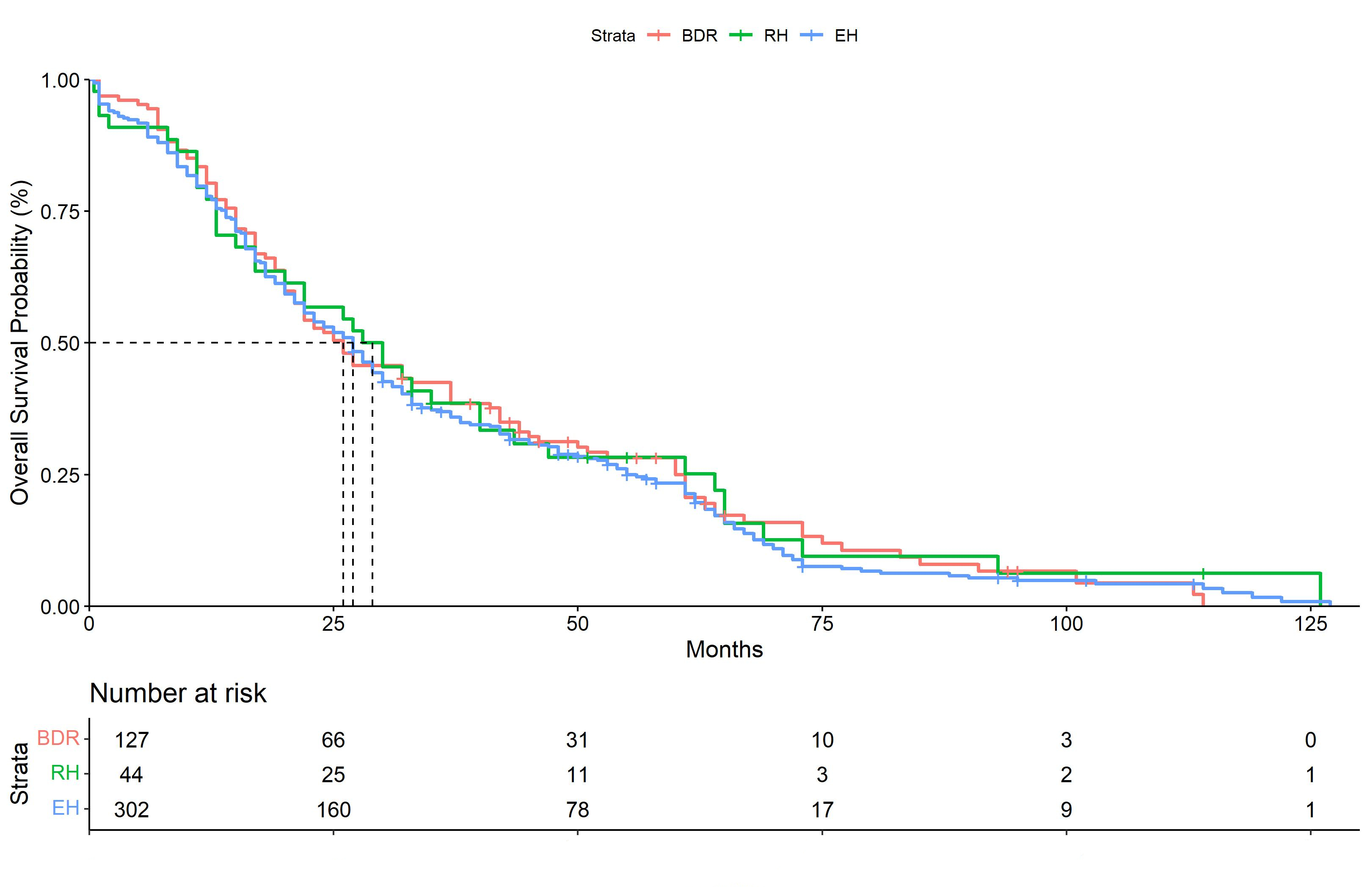


Figure S1 Overall survival analysis of patients with BDR, RH and EH

Abbreviations: BDR, bile duct resection; RH, restrictive hepatectomy; EH, extensive hepatectomy.


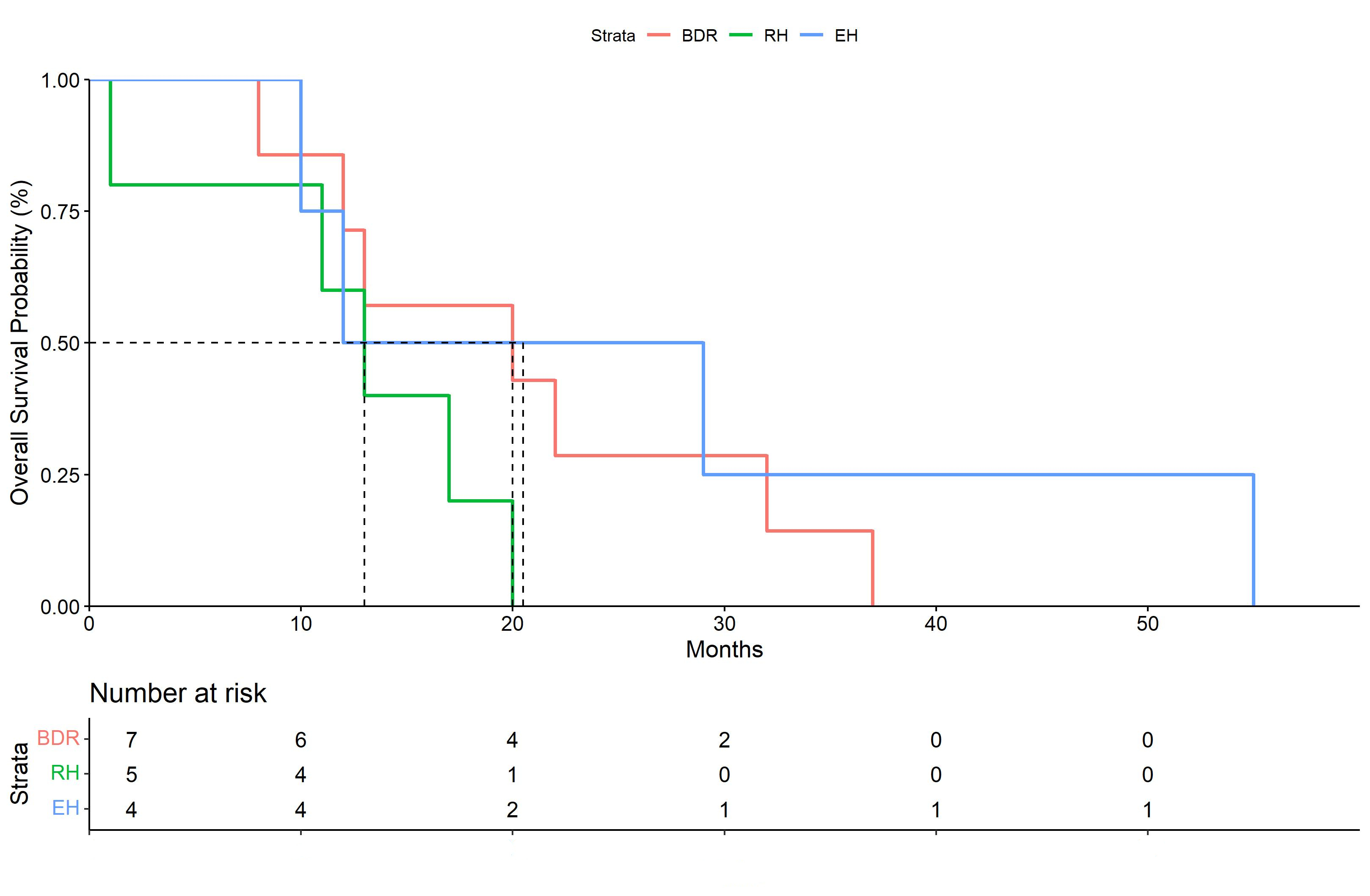


**Figure S2 Overall survival analysis of patients at TNM stage Ⅲa**

Abbreviations: BDR, bile duct resection; RH, restrictive hepatectomy; EH, extensive hepatectomy.


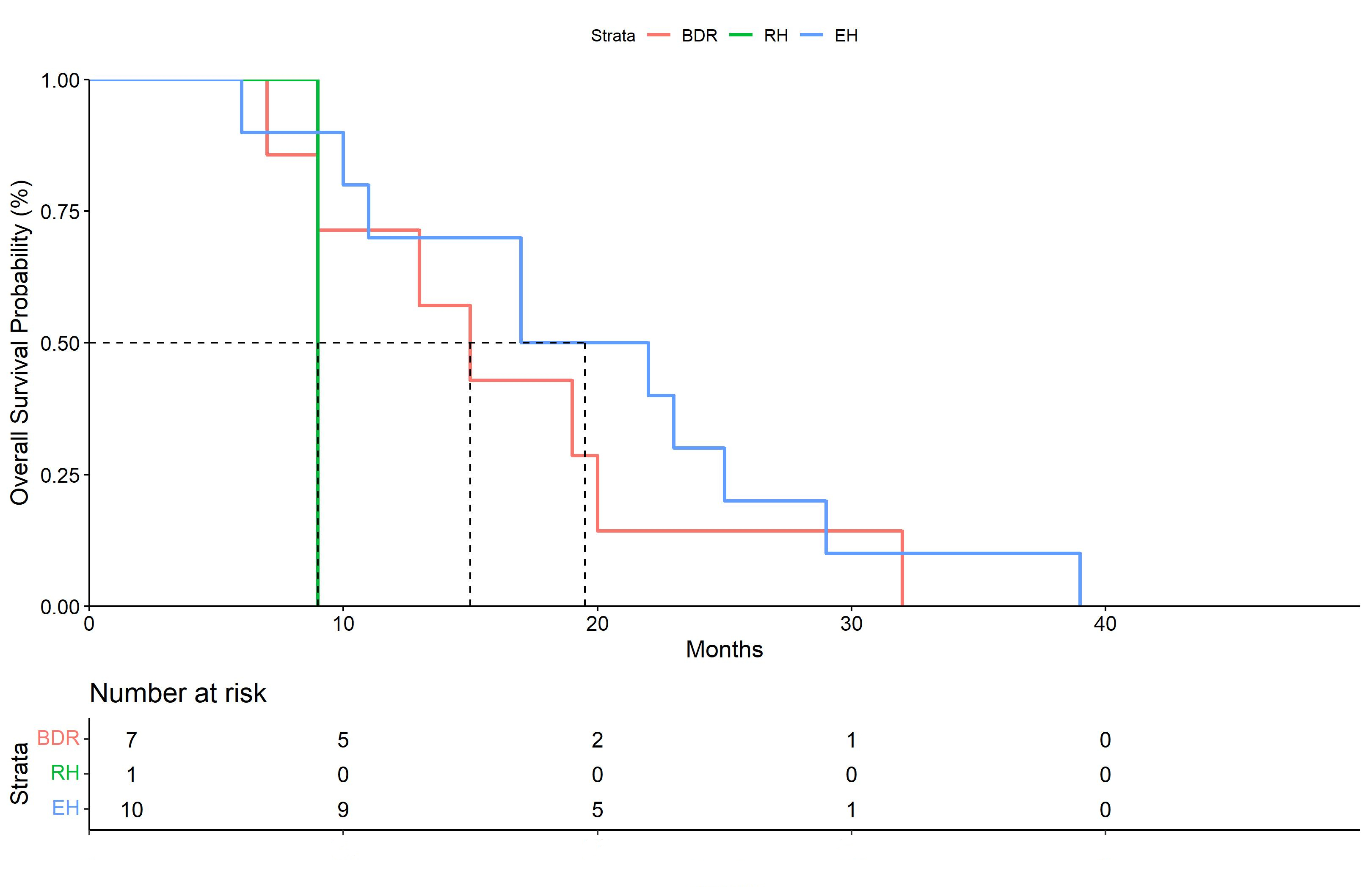


**Figure S3 Overall survival analysis of patients at TNM stage Ⅳ**

Abbreviations: BDR, bile duct resection; RH, restrictive hepatectomy; EH, extensive hepatectomy.
